# Mpox Outbreak — Los Angeles County, California, May 4–August 17, 2023

**DOI:** 10.15585/mmwr.mm7302a4

**Published:** 2024-01-18

**Authors:** Colleen M. Leonard, Kathleen Poortinga, Erin Nguyen, Abraar Karan, Sonali Kulkarni, Rebecca Cohen, Jacob M. Garrigues, Amy N. Marutani, Nicole M. Green, Andrea A. Kim,, Kwa Sey, Mario J. Pérez

**Affiliations:** ^1^Los Angeles County Department of Public Health, Los Angeles, California; ^2^Stanford University, Division of Infectious Diseases & Geographic Medicine, Stanford, California.

**Summary**

**What is already known about this topic?**

Mpox has disproportionately affected gay, bisexual, and other men who have sex with men. Vaccination against mpox has been shown to be protective against symptomatic mpox.

**What is added by this report?**

Mpox transmission occurred in Los Angeles County, California, during May–August 2023 at lower levels than in 2022 but at higher levels than during previous months and in other U.S. jurisdictions. Most mpox patients were not fully vaccinated. Two mild reinfections were reported.

**What are the implications for public health practice?**

Mpox continues to spread within Los Angeles County. This outbreak underscores the ongoing need for accessible mpox vaccination for persons at risk, particularly among young, Black or African American, and Hispanic or Latino persons, and persons living with HIV.

## Abstract

Since May 2022, approximately 2,500 mpox cases have been reported in Los Angeles County (LAC), California. Beginning in May 2023, the LAC Department of Public Health observed a consistent increase in mpox cases after a prolonged period of low incidence. A total of 56 cases were identified during May 4–August 17, 2023. A minority of mpox patients were fully vaccinated (29%). One patient was hospitalized; no deaths were reported. Two cases of reinfection occurred, both of which were associated with mild illness. The increasing number of cases during this period was significant, as few other health departments in the United States reported an increase in mpox cases during the same period. The outbreak spread similarly to the 2022 U.S. mpox outbreak, mainly through sexual contact among gay, bisexual, and other men who have sex with men. Vaccination against mpox became available in June 2022 and has been shown to be effective at preventing mpox disease. This outbreak was substantially smaller than the 2022 mpox outbreak in LAC (2,280 cases); possible explanations for the lower case count include increased immunity provided from vaccination against mpox and population immunity from previous infections. Nonetheless, mpox continues to spread within LAC, and preventive measures, such as receipt of JYNNEOS vaccination, are recommended for persons at risk of *Monkeypox virus* exposure.

## Epidemiologic Investigation and Findings

During May 4–August 17, 2023, a total of 56 laboratory-confirmed mpox cases occurred in Los Angeles County (LAC), based on illness onset date or laboratory specimen collection date (if onset date was missing) (Figure). In contrast, during the 3 months preceding May 4, 2023, only seven mpox cases were reported in LAC. In addition to requirements for laboratory reporting of all mpox tests, health care providers must report all mpox or orthopoxvirus infections and information on illness characteristics to the LAC Department of Public Health (LACDPH). LAC residents with laboratory-confirmed mpox were contacted for interview by a public health disease investigator to obtain information on demographic, epidemiologic, and clinical characteristics. Clinical information was obtained from a combination of self-report from interviews and the medical provider report from the patients’ provider. Among the 56 patients, 32 (57%) were unvaccinated, eight (14%) were partially vaccinated, and 16 (29%) were fully vaccinated.* All 56 cases occurred in persons who were assigned male sex at birth and who identified as male (Table). Overall, 45 (80%) mpox patients identified as gay or bisexual. The median patient age was 35 years (IQR = 26–42 years). Overall, 21 patients (38%) were non-Hispanic White (White) men, 18 (32%) were Hispanic or Latino (Hispanic), 13 (23%) were non-Hispanic Black or African American (Black), and four (7%) identified as another race. More than one half of patients (57%; 32) lived in the Los Angeles metropolitan area. Among 55 interviewed patients, 48 (87%) reported sexual contact in the 3 weeks preceding symptom onset. No common social events were reported. Two pairs of patients were epidemiologically linked (i.e., a patient disclosed sexual contact with another patient in the 3 weeks preceding symptom onset). Forty-two (76%) interviewed patients did not report any travel outside of LAC in the 3 weeks before symptom onset, suggesting local mpox transmission. This activity was reviewed by CDC, deemed not research, and was conducted consistent with applicable federal law and CDC policy.^†^

**FIGURE:**
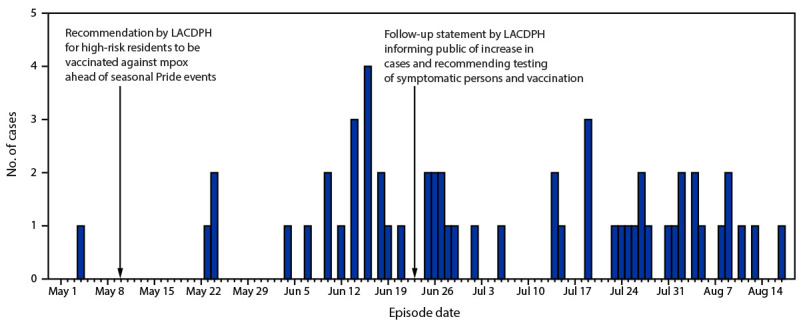
Laboratory-confirmed mpox cases, by episode date* (N = 56) — Los Angeles County, California, May 4–August 17, 2023 **Abbreviation:** LACDPH = Los Angeles County Department of Public Health. * Episode date is calculated from the symptom onset date or, if the symptom onset date is unknown, from specimen collection date.

**TABLE:** Characteristics of patients with mpox, by vaccination status — Los Angeles County, California, May 4–August 17, 2023

Characteristic	Vaccination status, No. (column %)*			
	All (N = 56)	Fully vaccinated (n = 16)	Partially vaccinated (n = 8)	Unvaccinated (n = 32)
**Median age, yrs (IQR)**	**35 (26–42)**	37 (31–46)	35 (25–45)	30 (26–38)
**Current gender identity**				
Male	**56 (100)**	16 (100)	8 (100)	32 (100)
**Sexual orientation**				
Gay or bisexual	**45 (80)**	14 (88)	6 (75)	25 (78)
Heterosexual	**6 (11)**	0 (—)	1 (13)	5 (16)
Other or unknown	**5 (9)**	2 (13)	1 (13)	2 (6)
**Race and ethnicity**				
Black or African American, non-Hispanic	**13 (23)**	0 (—)	1 (13)	12 (38)
White, non-Hispanic	**21 (38)**	12 (75)	3 (38)	6 (19)
Hispanic or Latino	**18 (32)**	3 (19)	3 (38)	12 (38)
Other	**4 (7)**	1 (6)	1 (13)	2 (6)
**Geographic area**				
Metro LA area	**32 (57)**	10 (63)	6 (75)	16 (50)
Outside metro LA	**24 (43)**	6 (37)	2 (25)	16 (50)
**Persons living with HIV**	**17 (30)**	5 (31)	3 (38)	9 (28)
CD4 >350	**14 (82)**	5 (100)	3 (100)	6 (67)
CD4 200–350	**3 (18)**	0 (—)	0 (—)	3 (33)
Not virally suppressed^†^	**6 (35)**	2 (40)	1 (33)	3 (33)
**Persons who are HIV-negative**	**39 (68)**	11 (69)	5 (63)	23 (72)
Receiving HIV PrEP^§^	**26 (67)**	11 (100)	4 (80)	11 (48)
**Persons hospitalized for mpox**	**1 (2)**	0 (—)	0 (—)	1 (3)
**Symptoms^¶^**				
Pruritis	**25 (46)**	7 (44)	4 (57)	14 (44)
Fever	**22 (40)**	5 (31)	2 (29)	15 (47)
Chills	**16 (29)**	3 (19)	2 (29)	11 (34)
Enlarged lymph nodes	**20 (37)**	5 (31)	3 (43)	12 (38)
Rectal bleeding	**11 (20)**	3 (19)	1 (14)	7 (22)
Lesions on the genital area	**36 (65)**	10 (63)	6 (86)	20 (63)
**Received tecovirimat**	**18 (32)**	7 (44)	2 (25)	9 (28)
Reported contact with someone with mpox^¶,^** symptoms	**7 (13)**	1 (6)	1 (14)	5 (16)
**Reported sexual contact 3 weeks before episode date** ^¶,††^	**48 (87)**	14 (88)	6 (86)	28 (88)
**Median no. of sex partners (range)** ^¶^ ^,^**	**2 (0–55)**	3 (0–10)	2 (0–3)	1 (0–55)
**Reported travel outside LA county ≤3 wks before episode date** ^¶,††^	**13 (24)**	5 (31)	2 (29)	6 (19)

**Abbreviations:** LA = Los Angeles; PrEP = preexposure prophylaxis.

* Some percentages might not sum to 100% because of rounding.

^†^ Last viral load result >200 copies/mL or no viral load result reported in previous 12 months.

^§^ At time of mpox case interview; all persons who were HIV-negative were interviewed.

^¶^ Fifty-five of 56 patients were interviewed, including all who were fully vaccinated, seven out of eight who were partially vaccinated, and all who were unvaccinated.

** Three weeks before symptom onset.

^††^ Episode date is calculated from the symptom onset date or specimen collection date if symptom onset is unknown.

### Demographic and Other Characteristics by Vaccination Status

Demographic and other patient characteristics were assessed by vaccination status (fully vaccinated with JYNNEOS vaccine, partially vaccinated, or unvaccinated). The median age of fully vaccinated patients was 37 years (IQR = 31–46 years), of partially vaccinated patients was 35 years (IQR = 25–45 years), and of unvaccinated patients was 30 years (IQR = 26–38 years). Black persons accounted for 23% of all cases; however, no Black patients were fully vaccinated. Likewise, whereas Hispanic persons accounted for approximately one third of all patients, only three of 18 were fully vaccinated at the time of infection. In contrast, among White patients, who accounted for 38% of all patients, 57% were fully vaccinated. Seventeen (30%) patients were living with HIV, five of whom were fully vaccinated. Three patients living with HIV had CD4 counts <350 cells/mm^3^; none of these patients was fully vaccinated. Among HIV-negative patients, 100% of those who were fully vaccinated were receiving HIV preexposure prophylaxis (PrEP) at time of interview, compared with 48% who were unvaccinated. Fully vaccinated patients reported more sex partners in the 3 weeks preceding symptom onset (median = three) than did those who were unvaccinated (one) or partially vaccinated (two).

### Previous Mpox Diagnosis

Two unvaccinated patients had previously received a diagnosis of mpox in 2022, 10 months and 12 months, respectively, before their 2023 infection. Review of clinical information confirmed that both patients had complete resolution of their previous infections before new symptom onset. Both secondary infections occurred in Black persons aged 35–45 years. One patient was living with HIV with a CD4 count <350 cells/mm^3^. Signs and symptoms in both patients with secondary infection were mild, and complete resolution was noted within the 3-week follow-up period.

### Mpox Severity

Data for fully assessing clinical severity according to the mpox severity scoring system (*1*) are incomplete; however, patient interviews indicated that most cases were mild. Compared with patients who were fully vaccinated with JYNNEOS vaccine, a larger proportion of those who were unvaccinated reported signs or symptoms of fever (47% versus 31%) and chills (34% versus 19%); other symptoms were similar irrespective of vaccination status. Eighteen (32%) patients, including seven who were fully vaccinated, received the antiviral drug tecovirimat to treat mpox symptoms. One HIV-negative patient with no immunocompromising conditions and who had not received any JYNNEOS vaccine was hospitalized for pain management and infectious disease evaluation while awaiting mpox laboratory test results.

### Mpox Vaccination History

Among the 16 fully vaccinated patients, the median interval from receipt of the second JYNNEOS vaccine dose to mpox symptom onset was 10 months (IQR = 9–11 months). Among fully vaccinated patients, six had received 2 subcutaneous vaccine doses and 10 had received 1 subcutaneous and 1 intradermal dose. Among the eight patients who had received 1 vaccine dose, five had received a subcutaneous dose and three had received an intradermal dose.

### Laboratory Investigation

Whole-genome sequencing followed by genomic analyses (*2*,*3*) were performed on outbreak specimens obtained from 45 patients (14 fully vaccinated, six partially vaccinated, and 25 unvaccinated) as part of LAC’s *Monkeypox virus* (MPXV) genomic surveillance program; specimens from three patients were sequenced by the California Department of Public Health. Phylogenetic analysis (*4*) (data set tag 2023–08–01T12:00:00Z) determined that 32 (71.1%) cases involved MPXV belonging to the B.1.20 lineage of clade IIb, which is currently the dominant lineage identified through surveillance in the United States. Twelve (26.7%) cases involved MPXV assigned a lineage of B.1 that formed a monophyletic group defined by four mutations relative to the B.1 reference genome (G70002A, G143951A, C148604T, and G154188A), and might represent an emerging sublineage. One case (2.2%) associated with travel to China involved MPXV belonging to the C.1 lineage, which is prevalent in East Asia. Recently, a tecovirimat-resistant MPXV variant was identified in LAC (*5*); however, mutations associated with tecovirimat resistance were not detected in any outbreak specimens.

## Public Health Response

On May 10, 2023, LACDPH issued a statement recommending that residents at high risk^§^ receive mpox vaccine ahead of seasonal Pride events.^¶^ LACDPH prepared for a potential increase in mpox cases before summer 2023 and supported mpox vaccinations at numerous on-site pop-up clinics at Pride events and festivals during May 1–August 31. During this period, 3,524 JYNNEOS vaccine first doses and 1,660 second doses were administered in LAC. On June 23, 2023, LACDPH released a follow-up statement notifying the public of the rise in cases and encouraging testing for persons with symptoms and vaccination for populations at high risk.

Public health disease investigators interviewed and followed up with patients who had received an mpox diagnosis to assess their disease progression and provide support. Among the 56 patients, 55 (98%) were reached for interview. Public health disease investigators also elicited close contact information and performed follow-up, offering postexposure prophylaxis to contacts when indicated. However, most patients reported sexual contact in the 3 weeks before symptom onset, but few patients disclosed names of their contacts. Although it is not unusual for persons with sexually transmitted infections to have a low contact index (the number of sex partners for whom information is sufficient to initiate contact efforts divided by the number of persons interviewed), this circumstance limited the ability to control mpox transmission through contact tracing efforts.

## Discussion

Mpox is spreading at low levels within LAC, which could be indicative of endemic transmission, especially as vaccination rates decline. The outbreak described in this report includes a smaller number of mpox cases (average of one case per day) compared with the number reported during the summer 2022 LAC outbreak, when an average of 39 cases per day were reported during the outbreak’s August 2022 peak (*6*). Vaccination against mpox has been shown to be an effective measure to prevent mpox disease, with the highest protection provided after receipt of 2 doses of JYNNEOS vaccine (*7*,*8*). The substantially lower number of cases in the 2023 outbreak could be due to increased immunity provided by vaccination against mpox or population immunity from previous infections. Fewer than one third of cases in this outbreak (29%) occurred in persons who were fully vaccinated, in contrast to the 2023 Chicago mpox outbreak (March–June) in which the majority of cases (55%) occurred in persons who were fully vaccinated (*9*). Similar to the 2023 Chicago mpox outbreak, all LAC patients identified as male (93% for Chicago), and the majority of patients were gay or bisexual (80% for both the LAC and Chicago outbreaks). In addition, the majority of patients in both outbreaks experienced self-limited illness that was managed in outpatient facilities. Genetic sequencing results from a subset of four cases in the Chicago outbreak identified MPXV among patients to be consistent with the B.1 lineage of clade IIb MPXV, similar to most of the cases from the LAC outbreak (44 of 45).

Mpox vaccination is recommended for all persons at risk, including men who have sex with men who have more than one sex partner and persons living with HIV (*10*). Only approximately 18% of persons living with HIV in LAC are fully vaccinated against mpox (LACDPH, unpublished data, October 2023). Although mpox vaccines are free (*10*), this outbreak underscores the ongoing need for accessible mpox vaccination for persons at risk for severe disease, particularly persons with uncontrolled or advanced HIV disease, and groups with low vaccination coverage, including young, Black, and Hispanic persons, and persons living with HIV. Better understanding of reasons for low vaccination rates could help increase coverage; alternative vaccination strategies for persons living with HIV might be needed at a time when the number of HIV specialty medical visits is declining because of the effectiveness of HIV antiretroviral therapy. Other measures, such as limiting the number of one’s sex partners, can also help prevent mpox transmission.

### Implications for Public Health Practice

Detection of this outbreak relied on the ability to detect cases through provider and laboratory reporting of laboratory-confirmed mpox. With the continued spread of mpox, health care providers should recommend vaccination to all persons at risk (*10*). Providers should remain aware of the ongoing transmission of MPXV, even among persons with previous infection or who have been vaccinated. Local mpox surveillance remains critical to differentiating whether mpox is causing more severe disease or spreading by new routes and to new risk groups.

## Footnotes

* Full vaccination was defined as receipt of ≥2 doses of JYNNEOS vaccine ≥24 days apart, with the second dose received ≥2 weeks before mpox episode date (illness onset date or laboratory specimen collection date [if onset date was missing]). Partial vaccination was defined as receipt of ≥1 dose of JYNNEOS vaccine ≥2 weeks before illness onset but fully vaccinated criteria was not met. Unvaccinated was defined as no evidence of vaccination before illness onset, or illness onset <2 weeks after receipt of the first vaccine dose.

† 45 C.F.R. part 46.102(l) (2), 21 C.F.R. part 56; 42 U.S.C. Sect. 241(d); 5 U.S.C. Sect. 552a; 44 U.S.C. Sect. 3501 et seq.

§ Persons at high risk were defined as 1) men or transgender persons who have sex with men or transgender persons, 2) persons of any gender or sexual orientation who have sex or intimate physical contact with others in association with a large event or engage in commercial or transactional sex, 3) persons living with HIV, especially those with uncontrolled or advanced HIV, or 4) sexual partners of persons belonging to any of the previous groups.

¶
http://publichealth.lacounty.gov/phcommon/public/media/mediapubhpdetail.cfm?prid=4380
